# Leptin potentiates *Prevotella intermedia* lipopolysaccharide-induced production of TNF-α in monocyte-derived macrophages

**DOI:** 10.5051/jpis.2010.40.3.119

**Published:** 2010-06-25

**Authors:** Sung-Jo Kim

**Affiliations:** Department of Periodontology, Pusan National University College of Dentistry, Yangsan, Korea.

**Keywords:** Leptin, Lipopolysaccharide, *Prevotella intermedia*, Tumor necrosis factor-α

## Abstract

**Purpose:**

In addition to regulating body weight, leptin is also recognized for its role in the regulation of immune function and inflammation. The purpose of this study was to investigate the effect of leptin on *Prevotella (P.) intermedia* lipopolysaccharide (LPS)-induced tumor necrosis factor (TNF)-α production in differentiated THP-1 cells, a human monocytic cell line.

**Methods:**

LPS from *P. intermedia* ATCC 25611 was prepared by the standard hot phenol-water method. THP-1 cells were incubated in the medium supplemented with phorbol myristate acetate to induce differentiation into macrophage-like cells. The amount of TNF-α and interleukin-8 secreted into the culture medium was determined by enzyme-linked immunosorbent assay (ELISA). TNF-α and Ob-R mRNA expression levels were determined by semi-quantitative reverse transcription-polymerase chain reaction analysis.

**Results:**

Leptin enhanced *P. intermedia* LPS-induced TNF-α production in a dose-dependent manner. Leptin modulated *P. intermedia* LPS-induced TNF-α expression predominantly at the transcriptional level. Effect of leptin on *P. intermedia* LPS-induced TNF-α production was not mediated by the leptin receptor.

**Conclusions:**

The ability of leptin to enhance *P. intermedia* LPS-induced TNF-α production may be important in the establishment of chronic lesion accompanied by osseous tissue destruction observed in inflammatory periodontal disease.

## INTRODUCTION

Periodontal disease is a chronic inflammatory process accompanied by destruction of surrounding connective tissue and alveolar bone, and sometimes loss of teeth [1]. The primary causative agents of periodontal disease are particular gram-negative anaerobic bacteria that accumulate in the gingival sulcus. *Prevotella (P.) intermedia* is a major periodontal pathogen that is dominant in the periodontal pockets of patients with adult periodontitis [2]. This bacterium has also been frequently recovered from subgingival flora in patients with acute necrotizing ulcerative gingivitis [3] and pregnancy gingivitis [4].

Lipopolysaccharide (LPS) is a major constituent of the outer membrane of gram-negative bacteria, including *P. intermedia*. It has the ability to trigger a number of host cells, especially mononuclear phagocytes, to produce and release a wide variety of pharmacologically active mediators, including interleukin (IL)-1β, IL-6, IL-8, and, most importantly, tumor necrosis factor alpha (TNF-α) [5].

Leptin, the 16-kDa nonglycosylated protein encoded by the *ob* gene, is synthesized mainly by adipocytes and plays a crucial role in the homeostasis of body weight by regulating food intake and energy expenditure, through its action on the hypothalamus [6-8]. In addition to regulating body weight, leptin is also recognized for its role in the regulation of angiogenesis [9], hematopoiesis [10], reproduction [11], immune function [12], and most importantly, inflammation [13].

Leptin has been classified as a cytokine because it shows tertiary structure similar to the class of long-chain helical cytokines that includes IL-6, IL-11, and leukemia inhibitory factor [14]. The biological activities of leptin are mediated through the interaction with its specific cell surface-leptin receptor, Ob-R, which exists in multiple isoforms sharing the same extracellular domain but differing in the length of transmembrane coding regions [12,15]. Leptin and Ob-R have been identified in various tissues and organs including the hypothalamus, adipose tissue, and gastric and intestinal mucosa, as well as in oral mucosa and the acinar cells of salivary glands [15-18]. The presence of leptin and Ob-R in oral mucosa and salivary glands suggests that the activity of leptin is also of significance to diseases affecting the oral cavity. The purpose of this study was to investigate the effect of leptin on *P. intermedia* LPS-induced TNF-α production in differentiated THP-1 cells, a human monocytic cell line.

## MATERIALS AND METHODS

### Bacteria and culture conditions

*P. intermedia* ATCC 25611 was used throughout. It was grown anaerobically on the surface of enriched Trypticase soy agar containing 5% (v/v) sheep blood, or in GAM broth (Nissui, Tokyo, Japan) supplemented with 1 µg/mL menadione and 5 µg/mL hemin. Culture purity was assessed by gram staining and plating on solid medium.

### LPS isolation

LPS was prepared from lyophilized *P. intermedia* ATCC 25611 cells by the standard hot phenol-water method [19], and subsequently purified by treatment with nuclease and proteinase K. The yield of LPS was about 0.26%. The protein content of the purified LPS, determined by the method of Markwell et al. [20], was less than 0.1%. Coomassie blue staining of overloaded sodium dodecyl sulfate (SDS)-polyacrylamide gels did not reveal any visible protein bands in the purified LPS, confirming the purity of the preparation (data not shown).

### Cell cultures

The human monocytic cell line THP-1 (American Type Culture Collection, Rockville, USA) was grown routinely in Nunc flasks in RPMI 1640 medium supplemented with 10% (v/v) heat inactivated FBS, 50 µM 2-mercaptoethanol, 1 mM sodium pyruvate, 25 mM HEPES, 100 U/mL of penicillin, and 100 µg/mL of streptomycin in a humidified chamber with 5% CO_2_/95% air at 37℃. The cells (5 × 10^5^ cells/ml/well in 24-well culture plates) were incubated in the medium supplemented with 50 ng/mL of phorbol myristate acetate to induce differentiation into macrophage-like cells. The cells were allowed to differentiate and adhere to plastic for 72 hours and washed three times with medium. Various concentrations of *P. intermedia* LPS and leptin (R & D Systems, Minneapolis, USA) were then added and the cells were cultured for the indicated times, after which culture supernatants were collected and assayed for TNF-α.

### Measurement of TNF-α and IL-8 production

The amount of TNF-α and IL-8 secreted into the culture medium was determined by enzyme-linked immunosorbent assay (ELISA) using a commercially available kit (OptEIA, BD Pharmingen, San Diego, USA) according to protocols recommended by the manufacturer. The sensitivity of the assay was 7.8 pg/mL, according to the manufacturer.

### Reverse transcription-polymerase chain reaction (RT-PCR) and analysis of PCR products

Cells were plated in 100-mm tissue culture dishes, at a density of 5 × 10^6^ cells per dish, and treated with various concentrations of *P. intermedia* LPS and leptin (R & D Systems, Minneapolis, USA) for the indicated periods of time. Following incubation, they were washed twice with phosphate buffered saline and collected by centrifugation. Total RNA was isolated with an RNeasy Mini Kit (Qiagen, Valencia, USA), according to the manufacturer's instructions. Synthesis of cDNA from the extracted RNA and subsequent amplification of the cDNA by RT-PCR were carried out with an AccuPower RT/PCR Premix kit (Bioneer, Seoul, Korea) and thermal cycler (GeneAmp PCR system 2400, PE Applied Biosystems, Foster City, USA). β-actin served as internal control. The number of cycles that ensured nonsaturating PCR conditions was established in preliminary experiments. Primer sequences and RT-PCR conditions are listed in Table 1. The PCR-amplified products were run on a 1.5% agarose gel containing ethidium bromide and visualized with UV light. The intensities of the PCR bands on gel photographs were quantified by densitometry.

### Statistical analysis

Statistical analysis was performed using Student's paired *t*-test with *P*<0.05 considered statistically significant. Data are expressed as means±SD of three independent experiments.

## RESULTS

### Effect of leptin on production of TNF-α and IL-8

**Figure 1:**
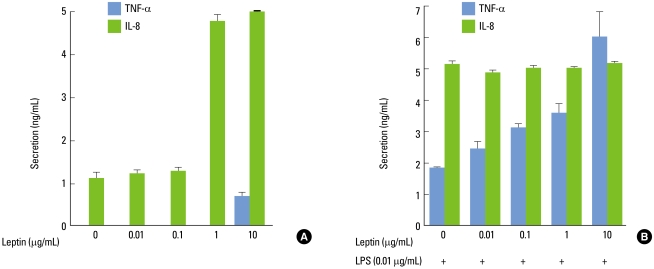
Effect of leptin on the release of tumor necrosis factor (TNF)-α and interleukin (IL)-8 by differentiated THP-1 cells. Cells were incubated with increasing concentrations of leptin (0.01-10 µg/mL) in the absence (A) or presence (B) of *Prevotella intermedia* lipopolysaccharide (LPS) (0.01 µg/mL). Supernatants were removed after 24 hours and assayed for TNF-α and IL-8. The results are means±SD of three independent experiments.

As shown in Fig. 1A, leptin induced a weak increase in TNF-α production in differentiated THP-1 cells, which became significant at 10 µg/mL. Moreover, *P. intermedia* LPS-induced TNF-α production was enhanced by leptin in a dose-dependent manner with a significant effect already seen at 0.01 µg/mL compared with LPS alone (Fig. 1B). Leptin at its concentration of 10 µg/mL elicited up to 3.25-fold increase in *P. intermedia* LPS-induced TNF-α production. Leptin increased IL-8 production without LPS with a significant effect at more than 1 µg/mL (Fig. 1A). However, *P. intermedia* LPS-induced IL-8 production was unaltered by leptin (Fig. 1B).

### Effect of leptin on TNF-α mRNA expression

**Figure 2:**
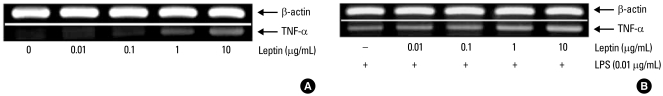
Effect of leptin on the expression of tumor necrosis factor (TNF)-α mRNA in differentiated THP-1 cells. Cells were incubated with increasing concentrations of leptin (0.01-10 µg/mL) in the absence (A) or presence (B) of *Prevotella intermedia* lipopolysaccharide (LPS) (0.01 µg/mL). See MATERIALS AND METHODS for further details. The polymerase chain reaction bands on a gel photograph in one of two separate experiments yielding similar results are shown.

To investigate whether leptin modulates *P. intermedia* LPS-induced TNF-α production at the gene level, TNF-α mRNA levels were determined by semi-quantitative RT-PCR analysis. As shown in Fig. 2A, leptin induced a concentration-dependent increase in TNF-α mRNA expression in differentiated THP-1 cells, which became significant at 1 µg/mL. Moreover, leptin potentiated *P. intermedia* LPS-induced expression of TNF-α mRNA in a dose-dependent manner (Fig. 2B).

### Effect on leptin receptor expression

**Figure 3:**
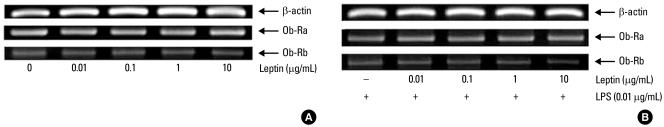
Effect of leptin on the expression of Ob-Ra and Ob-Rb mRNA in differentiated THP-1 cells. Cells were incubated with increasing concentrations of leptin (0.01-10 µg/mL) in the absence (A) or presence (B) of *Prevotella intermedia* lipopolysaccharide (LPS) (0.01 µg/mL). See MATERIALS AND METHODS for further details. The polymerase chain reaction bands on a gel photograph in one of two separate experiments yielding similar results are shown.

To verify whether the Ob-R is responsible for mediating the synergistic effect of leptin on *P. intermedia* LPS-induced TNF-α production in differentiated THP-1 cells, Ob-R mRNA expression levels were determined by semi-quantitative RT-PCR analysis. The long (Ob-Rb) and short (Ob-Ra) leptin receptors were expressed in untreated and leptin-treated cells, while upregulation of Ob-R by *P. intermedia* LPS stimulation or its potentiation by leptin was not confirmed (Fig. 3). As shown in Fig. 3, the levels of expression of the leptin receptors were similar in all samples.

## DISCUSSION

Because production of TNF-α has been recognized as a marker in a variety of human diseases associated with inflammation [21], the effect of leptin on *P. intermedia* LPS-induced TNF-α production in differentiated THP-1 cells, a human monocytic cell line, was studied. Macrophages are known to be the main producer of TNF-α and a dense infiltration of inflammatory cells, including macrophages, occurs in the gingival connective tissues of patients with periodontal disease [22].

Leptin levels are increased by inflammatory stimuli such as LPS and cytokines [23-25]. And, leptin enhances pro-inflammatory cytokine production and phagocytosis by macrophages [26]. The plasma levels of leptin have been reported to increase as periodontal disease progressed [27]. Conversely, it was shown that the leptin concentration is higher in healthy gingival tissue than in diseased tissue [28]. Further, the results of Karthikeyan and Pradeep [27] showed a strong negative correlation between the GCF leptin concentration and periodontal disease progression. Their results are in accordance with the study done by Johnson and Serio [29], who also showed that leptin concentration is correlated negatively with the probing pocket depth. The mechanism underlying the above findings is not known. Leptin may be used up as a substrate during inflammation.

The results of this study indicate that leptin potentiates *P. intermedia* LPS-induced TNF-α production in a dose-dependent manner. Leptin modulated *P. intermedia* LPS-induced TNF-α expression predominantly at the transcriptional level. There is evidence to suggest that TNF-α plays a central role in the pathogenesis of periodontal disease. TNF-α has been found at high levels in gingival crevicular fluids and in gingival tissues from periodontally diseased sites over those in healthy sites [30,31]. Moreover, it was shown that TNF-α has a strong potential to induce connective tissue degradation and alveolar bone resorption [32,33]. Furthermore, blockade of the activity of TNF-α was found to inhibit the inflammatory response and bone loss in a primate model of experimental periodontitis [34]. The ability of leptin to enhance *P. intermedia* LPS-induced TNF-α production may be important in the establishment of chronic lesion accompanied by osseous tissue destruction observed in inflammatory periodontal disease.

To verify whether the mechanism behind the synergistic effect of leptin with *P. intermedia* LPS might be related to an increase in Ob-R expression, Ob-R mRNA expression levels were determined by semi-quantitative RT-PCR analysis. This study demonstrated the presence of the long and short leptin receptors in differentiated THP-1 cells. However, leptin receptors were not modulated by *P. intermedia* LPS or leptin. These results indicate that effect of leptin on *P. intermedia* LPS-induced TNF-α production is not mediated by the leptin receptor. The mechanism by which leptin potentiates *P. intermedia* LPS-induced TNF-α production remains to be elucidated.

## Figures and Tables

**Table 1:**
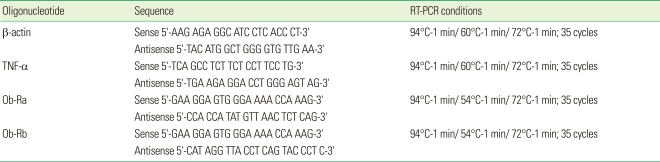
Nucleotide sequences of probes used in RT-PCR assays and amplification conditions.

RT-PCR: reverse transcription-polymerase chain reaction, TNF: tumor necrosis factor.
